# COMT Val^158^Met genotypes differentially influence subgenual cingulate functional connectivity in healthy females

**DOI:** 10.3389/fnhum.2014.00481

**Published:** 2014-06-30

**Authors:** Chris Baeken, Daniele Marinazzo, Stephan Claes, Guo-Rong Wu, Peter Van Schuerbeek, Johan De Mey, Robert Luypaert, Rudi De Raedt

**Affiliations:** ^1^Department of Psychiatry and Medical Psychology, Ghent UniversityGhent, Belgium; ^2^Department of Psychiatry, University Hospital (UZBrussel)Brussels, Belgium; ^3^Ghent Experimental Psychiatry (GHEP) Lab, Ghent UniversityGhent, Belgium; ^4^Department of Data Analysis, Ghent UniversityGhent, Belgium; ^5^Department of Psychiatry, University Hospital (UZLeuven)Leuven, Belgium; ^6^Key Laboratory of Cognition and Personality, Faculty of Psychology, Southwest UniversityChongqing, China; ^7^Department of Radiology and Medical Imaging, University Hospital (UZBrussel)Brussels, Belgium; ^8^Department of Experimental Clinical and Health Psychology, Ghent UniversityGhent, Belgium

**Keywords:** sgACC, functional connectivity, COMT, females, genotype differences

## Abstract

Brain imaging studies have consistently shown subgenual Anterior Cingulate Cortical (sgACC) involvement in emotion processing. catechol-O-methyltransferase (COMT) Val^158^ and Met^158^ polymorphisms may influence such emotional brain processes in specific ways. Given that resting-state fMRI (rsfMRI) may increase our understanding on brain functioning, we integrated genetic and rsfMRI data and focused on sgACC functional connections. No studies have yet investigated the influence of the COMT Val^158^Met polymorphism (rs4680) on sgACC resting-state functional connectivity (rsFC) in healthy individuals. A homogeneous group of 61 Caucasian right-handed healthy female university students, all within the same age range, underwent rsfMRI. Compared to Met^158^ homozygotes, Val^158^ allele carriers displayed significantly stronger rsFC between the sgACC and the left parahippocampal gyrus, ventromedial parts of the inferior frontal gyrus (IFG), and the nucleus accumbens (NAc). On the other hand, compared to Val^158^ homozygotes, we found in Met^158^ allele carriers stronger sgACC rsFC with the medial frontal gyrus (MFG), more in particular the anterior parts of the medial orbitofrontal cortex. Although we did not use emotional or cognitive tasks, our sgACC rsFC results point to possible distinct differences in emotional and cognitive processes between Val^158^ and Met^158^ allele carriers. However, the exact nature of these directions remains to be determined.

## Introduction

Brain imaging studies point to functional differences between catechol-O-methyltransferase (COMT) Val^158^Met carriers in the prefrontal and limbic areas related to emotional processing (Rasch et al., 2010; Shehzad et al., 2012). Whereas Met^158^ allele carriers may have higher risk of developing mood and anxiety disorders (Hosák, 2007; Kocabas et al., 2010; Witte and Flöel, 2012), individuals carrying the Val^158^ variant are thought to display more cognitive difficulties (Swart et al., 2011). However, these assumptions are not yet fully established and reverse findings have been reported (Amstadter et al., 2012). Indeed, some studies reported that the Met^158^ allele is associated with increased limbic responsiveness to negative stimuli (Smolka et al., 2005, 2007), whereas others reported the opposite (Kempton et al., 2009; Domschke et al., 2012) or described null-results (Drabant et al., 2006). Further, the meta-analysis of Mier et al. (2010) provided evidence for a neural prefrontal cortical substrate of the pleiotropic behavioral effects of COMT Val^158^Met (rs4680) genetic variation. This means that a single gene has an effect on the expression of two or more phenotypic traits; in this case the COMT Val^158^Met gene that aligns with a differential impact on cognitive and emotional function. These opposing effects were found for executive cognition paradigms (favoring Met^158^ allele carriers) and emotional paradigms (favoring the Val^158^ variant). To summarize, at the brain level it remains poorly understood how these Val^158^Met single nucleotide polymorphisms (SNP) interfere with cognitive and/or emotion associated neurocircuits.

Resting state fMRI (rsfMRI)—used to measure neuronal connections between distinct regions, referred to as resting-state functional connectivity (rsFC; Biswal et al., 1995)–may have the potential to gain more insight in the genetic influence on these neuronal processes (Fox et al., 2012). More in particular, rsFC has several practical and theoretical advantages over task based fMRI, including improved signal to noise, a reduced need for participant compliance, and the avoidance of task performance confounds (Fox and Greicius, 2010). However, until now, only two brain imaging studies applied rsFC together with the COMT Val^158^Met gene typing. Tunbridge et al. (2013) showed that Val^158^ homozygotes displayed greater rsFC than the Met^158^ allele carriers between an executive control network and the ventrolateral parts of the prefrontal cortex during cognitive task performance (working memory). These findings concur with the observations of Liu et al. (2010) on default network connectivity where the Val^158^ homozygotes compared to COMT Val^158^ and Met^158^ heterozygous individuals also showed poor cognitive performance, possibly through differential effects on prefrontal dopamine levels. Indeed, homozygote Val^158^ carriers display higher enzymatic activity resulting in less prefrontal dopamine, whereas for the Met^158^ variant carriers this is the reverse (Heinz and Smolka, 2006). Further, stress-related influences may affect COMT Val^158^ and Met^158^ allele carriers differently (Antypa et al., 2013). Under stress, working memory performance of Met^158^ homozygotes was significantly worse compared to Val^158^ homozygotes (Buckert et al., 2012). Carriers of the Val^158^ allele of COMT (rs4680) were also more likely to quit a task measuring the level of distress intolerance than those without a Val^158^ allele (Amstadter et al., 2012). This is in line with the assumption that the Val^158^ allele is associated with cognitive inefficiency during tasks involving cognitive control. Bishop et al. (2006) found that, during the performance of a house-matching task under emotional distraction, the Val^158^ load correlated positively with activity in parahippocampal regions.

Importantly, dopaminergic activity within the ventromedial prefrontal cortex (vmPFC), including the subgenual anterior cingulate cortex (sgACC), has been associated with subjective levels of psychosocial stress (Lataster et al., 2011). This ACC region is part of distributed corticolimbic neurocircuits implicated in “visceromotor” functions and in modulating affect, such as sadness and ruminative thought patterns (Disner et al., 2011; Smith et al., 2011; Davey et al., 2012). The sgACC also participates in cognitive functions that create sad moods and lead to pessimism (Meyer, 2012).

Because the sgACC is a key region in modulating emotional behavior (Drevets et al., 2008), our main research objective was to examine whether in the Val^158^Met SNP (rs4680) differently influences sgACC rsFC with areas related to emotion and/or cognitive functioning. Because age (Ferreira and Busatto, 2013) and gender (Wang et al., 2014), may confound rsFC results, and given that age and gender influences have also been reported for the COMT Val^158^Met gene (Harrison and Tunbridge, 2008; Witte and Flöel, 2012), all female participants were selected within a narrow age range and were documented never to have suffered from neuropsychiatric illnesses. The chosen sgACC seed Montreal Neurological Institute (MNI) coordinates were based on brain anatomical coordinates provided by a neuroimaging study of emotion processing and emotion regulation in women resilient or susceptible to the depressogenic effects of early life stress (Cisler et al., 2013).

In Val^158^ carriers we hypothesized predominantly sgACC rsFC correlations with anatomical areas related with cognitive functioning. In Met^158^ carriers we expected sgACC rsFC associations with neurocircuits implicated in emotional processing. Of note, at this stage we cannot draw hypotheses about the direction of these correlations.

## Materials and methods

### Participants

The study was approved by the ethics committee of the University Hospital (UZBrussel) and all subjects gave written informed consent. It was part of a larger project for investigating different neuro-cognitive markers in affective disorders, in which a total of 80 healthy females were recruited. All participated in another study which evaluated the influence of the COMT Val^158^Met gene on personality dimensions. These results are published elsewhere (Baeken et al., in press).

Sixty-one right-handed Caucasian female participants, all university students (mean age = 21.8 years, sd = 2.5 years), participated in the rsfMRI study. Right-handedness was assessed with the van Strien questionnaire (van Strien and Van Beek, 2000). No subjects had ever used major psychotropic medications and at the time of the experiments all were free of any drug or medication, other than birth-control pills. To exclude psychiatric or neurological diseases all volunteers were screened by the first author (Chris Baeken). Psychiatric disorders were assessed by the Dutch version of the (MINI; Sheehan et al., 1998). Subjects with a psychiatric disorder and/or a score higher than eight on the Beck Depression Inventory (BDI-II; Beck et al., 1996) were excluded.

### Genetics

In a first step, after the rsfMRI scan EDTA acid anti-coagulated blood samples were drawn from each participant and DNA was isolated. In a second step, genotyping of COMT rs4680 SNP was performed using the MassARRAY platform (SEQUENOM, San Diego, CA).

### Scanning procedure

All participants were instructed to stay awake with their eyes closed and to think of nothing in particular during the resting state measurements, involving exactly 5 min of scanning. To reduce sensory confounds as much as possible, the light in the room was dimmed during scanning. After the scan, the subjects were asked to confirm that they had been awake throughout the scan and had complied with the instructions. All rsfMRI scans were performed on Monday afternoons, between 15:00 and 18:00.

To obtain individual anatomical information, all subjects underwent a T1-weighted MRI of the brain (3D-TFE, voxel size 1 × 1 × 1 mm) using a 3T Achieva MR scanner with an eight-channel SENSE head coil (Philips, Best, The Netherlands). The rsfMRI used a SE-EPI sequence (TR/TE = 3000/70 ms; flip angle = 90°; FOV = 230 × 230 mm^2^; resolution = 1.80 × 1.80 mm^2^; slice thickness/gap = 4.00/1.0 mm; number of slices = 24; number of dynamics = 100; time resolution = 3000 ms). After the rsfMRI scan an additional 3D anatomical scan using a 3D T1 TFE sequence (TR/TE = 12.00/3.71 ms; flip angle = 10°; FOV = 240 × 240 × 200 mm^3^; resolution = 1.00 × 1.00 × 2.0 mm^3^; number of slices = 100) was performed, yielding an anatomical underlay for the fMRI results.

The fMRI data were analyzed with the SPM8 software (Wellcome Department of Cognitive Neurology, London, UK). Slice-time correction was performed to correct for small differences in the time offset of consecutively measured slices. Hereafter, the images were realigned to the first volume of the time series in order to correct for head movements. Subsequently, all fMRI brain volumes were normalized to the EPI MNI template; resampled to 3-mm isotropic voxels and spatially smoothed using an 8-mm full-width half-maximum Gaussian kernel. The anatomical scans were normalized to the T1 MNI template.

Functional imaging data were linearly detrended and band-pass filtered (0.01–0.8 Hz). Spurious or nonspecific sources of variance were removed from the data through linear regression of: (1) the six head-motion parameters obtained in the realigning step; (2) the signal from a region in the cerebrospinal fluid; (3) the signal from a region centered in the white matter; and (4) the whole-brain signal. Correlation maps were obtained by extracting the BOLD time course from a seed region, then computing the correlation coefficients characterizing the correlations between that time course and the time courses from all other brain voxels. The seed region was a 6-mm-diameter sphere designed to encompass the (sgACC: *x* = 1, *y* = 25, *z* = −11), as provided by Cisler et al. (2013). We already used these seed sgACC coordinates in depressed patients (Baeken et al., 2014b), as well as the amygdalae seed coordinates in stress sensitive healthy females (Baeken et al., 2014a). Further, in a second step to increase the chance to get signal from functionally overlapping regions, we doubled the diameter of the sgACC sphere to 12 mm.

The correlation maps were submitted to a random-effect analysis in SPM8 following a one-way ANCOVA containing COMT (Val^158^–Val/Met^158^–Met^158^) as factor and age as covariate. These analyses were thresholded with Alphasim correction (residuals) as implemented in the SPM REST toolbox^1^ at *p* < 0.05. The result was a cluster extent threshold (K) of 46 voxels and a voxel significance threshold of 0.01. The anatomical labels and MNI coordinates were obtained by the xjView MATLAB toolbox.^2^ In a following step, to investigate whether the significant clusters corresponded to increased or decreased functional connectivity, *post-hoc* paired *t*-tests were performed in Marsbar (Brett et al., 2002). Paired variables were the contrast values for the three different groups (Val^158^–Val/Met^158^–Met^158^) separately. These paired *t*-tests were corrected for the number of ROIs. The significance level was set at *p* ≤ 0.05 (corrected), two-tailed.

## Results

**Figure 1:**
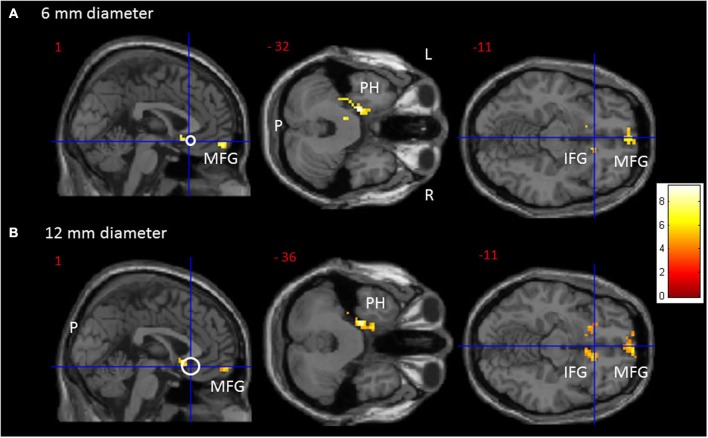
**Transversal and sagittal slides exhibiting significant rsFC clusters (red to yellow) for the sgACC seed (MNI: *x* = 1, *y* = 25, *z* = −11). (A)** For the 6 mm and **(B)** for the 12 mm diameter sgACC seed. The seed is displayed as a crosshair on an open white circle. CAVE: these white circles represent an estimate and not by definition a 1/1 scale. For an overview of all significant interaction clusters see Table 2. P = posterior, L = left, R = right. IFG = Inferior Frontal Gyrus, MFG = Medial Frontal Gyrus, PH = parahippocampus.

From the 61 participants, 14 were Val^158^ carriers, 37 Val/Met^158^ heterozygotes, and 10 were Met^158^ homozygotes. No volunteer stated to have fallen asleep during scanning. The calculation of the Hardy-Weinberg Equilibrium for two alleles showed no deviation of this assumption (*X*^2^_(1, *N* = 61)_ = 2.89, *p* = 0.09). A one-way ANOVA did not show age differences between the three groups (*F*_(2,58)_ = 0.62, *p* = 0.54). Significant one-way ANOVA sgACC rsFC clusters are displayed in Figure 1 for the 6 and 12 mm diameter seed sphere separately. For an overview of all significant clusters see also Table 1.

**Table 1:** **Results of the one-way ANCOVA for the sgACC rsFC containing COMT (Val^158^–Met^158^–Val/Met^158^) as factor and age as covariate for the 6 mm and radius 12 mm diameter sgACC seed**.

**sgACC Seed**	**Cluster size**	**Hemisphere**	**Anatomical region**	**BA**	***F*-value One-way ANOVA**	**Peak coordinates (x,y,z) (mm)**
**Diameter 6 mm**	63	Left	Parahippocampus	–	9.31	−18 −9 −33
	50	Right	Inferior Frontal Gyrus	BA 47	8.37	15 24 −15
	47	Left	Medial Frontal Gyrus	BA 11	7.72	0 60 −15
**Diameter 12 mm**	50	Left	Parahippocampus	–	9.88	−21 −15 −36
	120	Right	Anterior Cingulate	Caudate nucleus / BA 47	11.34	9 15 −9
	61	Left	Medial Frontal Gyrus	BA 11	7.81	−9 63 −15

*For each significant cluster, we report the *F*-value (peak intensity) and MNI coordinates (*x*, *y*, *z*) at the position of the maximum, the cluster size (k) and the corresponding Brodmann area (BA)*.

**Table 2:** **Results of the *post hoc* paired *t*-test from the sgACC rsFC one-way ANCOVA**.

**T-values**					
**sgACC seed**	**diameter 6 mm**			**diameter 12 mm**		
**COMT Val^158^Met gene**	**Parahippocampus**	**BA 47**	**BA 11**	**Parahippocampus**	**Caudate nucleus/BA 47**	**BA 11**
**Met > Val**	4.49	4.76	2.39 *	4.88	4.91	2.87 **
**Val/Met > Val**	0.98	1.70	4.76 **	1.59	4.42	1.39 **
**Val > Met**	4.49 **	4.76 **	2.39	4.88 **	4.91 **	2.87
**Val/Met > Met**	4.25 **	3.95 **	1.29	4.40 **	4.63 **	4.42
**Met > Val/Met**	4.25	3.95	1.29	4.40	4.63	0.89
**Val > Val/Met**	0.98	1.70	4.76	1.59	1.39	4.42

Clusters are listed (*p* value (corrected) < 0.05). For each cluster, we reported the T-value (peak intensity) at the position of the maximum and the corresponding Brodmann area (BA) for the 6 mm and 12 mm diameter sgACC seed. Significant *p* values: * *p* < 0.05, ** *p* < 0.01.

### Results confined to the 6 mm sgACC seed

The one-way ANOVA revealed three significant clusters with the first located in the left parahippocampal gyrus (MNI coordinates: *x* = −18, *y* = −9, *z* = −33) and the second in the right inferior frontal gyrus (IFG) (BA 47: *x* = 15, *y* = 24, *z* = −15). Here *post-hoc* testing showed significantly stronger rsFC between the sgACC and these clusters in Val^158^ and Val/Met^158^ carriers compared to Met^158^ carriers. The third interaction cluster was located in the left medial frontal gyrus (MFG) (BA 11: *x* = 0, *y* = 60, *z* = −15) and here the *post-hoc* test indicated a stronger sgACC rsFC with the left BA 11 in Met^158^ and Val/Met^158^ carriers compared to Val^158^ carriers. See Table 2.

### Results confined to the 12 mm sgACC seed

Here, the one-way ANOVA also revealed three significant clusters located in similar areas as before: the left parahippocampal gyrus (MNI coordinates: *x* = −21, *y* = −15, *z* = −36), the right IFG extending to the caudate nucleus (*x* = 9, *y* = 15, *z* = −9), and the left MFG (BA 11: *x* = −9, *y* = 63, *z* = −15). *Post-hoc* testing showed similar directions as found for the 6 mm sgACC seed for the Val^158^–Val/Met^158^–Met^158^ carriers. See also Table 2.

## Discussion

As hypothesized, we found different sgACC rsFC patterns in Val^158^ and Met^158^ carriers. Compared to the Met^158^ homozygotes, COMT Val^158^ and Val/Met^158^ carriers were found to exhibit stronger sgACC rsFC associations with the left parahippocampus (PH), the ventromedial parts of the inferior frontal cortex, and the nucleus accumbens (NAc) and caudate nucleus. The involvement of hippocampal areas has been demonstrated earlier in different brain imaging studies examining the effect of the COMT Val^158^Met polymorphism on cognitive processes, usually reporting that Val^158^ carriers perform worse on memory tasks than Met^158^ homozygotes (Bertolino et al., 2008; Heim et al., 2013); in particular in stressful situations (Buckert et al., 2012). As mentioned before, Val^158^ carriers recruited the parahippocampal regions while performing a house-matching task during emotional distraction, a typical attentional control related task (Bishop et al., 2006). Although we did not observe differences between homo-and heterozygote Val^158^ carriers and we did not include stress-related tasks (other than lying in the scanner with eyes closed), our findings support the hypothesis that COMT Val^158^ and Met^158^ carriers engage different neurocircuits while performing cognitive loaded tasks (Dennis et al., 2010). Indeed, because the sgACC serves as a facilitator of visceral responses during emotional processing–more in particular in stress-related conditions (Mayberg et al., 1999; Baeken et al., 2010)–the observed FC between the sgACC and the (left) PH in Val^158^ carriers could imply a stronger “emotional” participation associated with memory and recollection processes which may interfere with task achievement outcomes (Eichenbaum et al., 2007, 2012). Val^158^ carriers also displayed a stronger sgACC rsFC with the ventromedial IFG, which extended bilaterally to the NAc (which is part of the ventral striatum), and to the caudate nucleus (12 mm diameter sgACC seed), both areas comprising mostly dopaminergic neurons. This could be in line with the neurobiological action of the COMT Val^158^Met gene where Val^158^ carriers display higher enzymatic activity resulting in less prefrontal dopamine (Witte and Flöel, 2012). Again, as we did not include cognitive tasks and no direct dopaminergic measurements, these assumptions should be interpreted cautiously at this point. Because this is a *post-hoc* data driven interpretation on FC, additional data on the dopaminergic system will be crucial to verify that a stronger sgACC rsFC in Val^158^ carriers is related to dopamine enzymatic activity. Indeed, in spite that functional connectivity is a unique powerful tool able to increase our knowledge on human brain organization, rsFC is based on an inherently ambiguous measure reflecting constraints both from static anatomical connectivity and from poorly understood functional coupling changes that are dynamic; physiological data from other sources may be required to confirm and interpret FC findings (Buckner et al., 2013).

*Post-hoc* *t*-tests showed that compared to Val^158^ homozygotes, Met^158^ and Val/Met^158^ carriers displayed stronger sgACC rsFC with the MFG, more in particular the (left) orbitofrontal cortex (OFC: Brodmann area (BA) 11). Again, no differences between Met^158^ homo- and heterozygote allele carriers were observed. Other researchers also found OFC involvement in Met^158^ carriers related to emotional or arousal processing (Drabant et al., 2006; Montag et al., 2008; Dreher et al., 2009). Here, in contrast to the more lateral parts of the OFC, the medial OFC regions showed stronger connectivity with the default mode network, the limbic system, and areas related to autonomic processes. For a recent overview see Zald et al. (2014). Interestingly, it has recently been hypothesized by Falk et al. (2012) that Met^158^ allele carriers may be more sensitive to cues that signal social reward or punishment and that carrying the Met^158^ allele may be associated with greater neural activity to reward-related stimuli (Lancaster et al., 2012). This might predispose them to seek approval and thus exhibit more social conformity (Deuker et al., 2013). In line with these findings, a recent meta-analysis showed that medial OFC/vmPFC may subserve different roles in processing of reward magnitude (Diekhof et al., 2012). These dopaminergic neurons coming from the ventral tegmental area (VTA) are crucial for the recognition of rewards and their consumption or lack of consumption (Russo and Nestler, 2013). Although reverse findings have been reported (Hopkins et al., 2013), carrying the Met^158^ allele is associated with increased activity in limbic areas and prefrontal cortex. Of note, Met^158^homozygotes also exhibit greater anxiety (Montag et al., 2008; Baumann et al., 2013).

However, as our sample is relatively small, the interpretation of these results should be done cautiously. Due to the nature of this study, we can only draw conclusions regarding right-handed healthy young women. Although the selection of psychopathology-free female subjects within a narrow age-range can be considered as a major advantage of the study, including only healthy women within a certain age range does mean that we cannot generalize our findings to men, older women or subjects with any form of psychiatric illness. Because we did not *a priori* select our participants based on their genetic COMT Val^158^Met profile, the three groups were unbalanced, which might have influenced our imaging results. To some extent this may explain the lack of sgACC rsFC differences between COMT Val^158^Met homo- and heterozygotes. Another limitation of our study is the fact that no cardiac and respiratory data were collected during rsfMRI. Besides head movement, cardiac pulsation during scanning results in brain tissue movement and inflow effects leading to correlated signal fluctuations. Further, chest movement during breathing results in magnetic field changes that distort the MR image acquired of the brain (For an overview see Birn, 2012). Further, our hypothesis on sgACC involvement in the COMT Val^158^Met polymorphism was determined *a priori*. By not examining other dedicated seeds important information may have been missed. However, our main research objective was to examine the COMT Val^158^ Met gene in relation a specific node documented in women to be involved in emotion (dys)regulation in brain networks (Cisler et al., 2013), which makes the choice of the sgACC seed particularly relevant. Further, this seed based correlation analysis technique has been successfully applied by many research groups and has been proven useful in revealing the connectivity properties of similar seed areas (Cole et al., 2010). In addition, the test-retest reliability of such seed based correlation analysis has indicated moderate to high reliability to examine resting-state networks (Shehzad et al., 2009).

To summarize, in line with our hypotheses, we found distinct sgACC rsFC differences between Val^158^ and Met^158^ allele carriers. In spite that we did not use emotional or cognitive tasks, our sgACC rsFC results could represent specific neural substrates for the pleiotropic behavioral effects of COMT genetic variation between Val^158^ and Met^158^ allele carriers. Although our results corroborate an emotional influence on the COMT Val^158^Met polymorphism, future imaging research examining this SNP, also with dopaminergic brain measurements, may do well to include a larger number of seeds implicated in emotional and cognitive functioning.

## Conflict of interest statement

The authors declare that the research was conducted in the absence of any commercial or financial relationships that could be construed as a potential conflict of interest.

## Footnotes

^1^restfmri.net/forum/

^2^http://www.alivelearn.net/xjview
